# Ternary Restoration Binders as Piezoresistive Sensors: The Effect of Superplasticizer and Graphene Nanoplatelets’ Addition

**DOI:** 10.3390/nano15070538

**Published:** 2025-04-02

**Authors:** Maria-Evangelia Stogia, Ermioni D. Pasiou, Zoi S. Metaxa, Stavros K. Kourkoulis, Nikolaos D. Alexopoulos

**Affiliations:** 1 Research Unit of Advanced Materials, Department of Financial and Management Engineering, University of the Aegean, 82132 Chios, Greece; 2 Laboratory for Testing and Materials, Department of Mechanics, School of Applied Mathematical and Physical Sciences, Theocaris Building, National Technical University of Athens, Zografou Campus, 15780 Athens, Greece; 3 Hephaestus Laboratory, School of Chemistry, Faculty of Sciences, Democritus University of Thrace, 65404 Kavala, Greece

**Keywords:** graphene-based cementitious composites, lime–metakaolin nanocomposites, graphene nanoplatelets, electrical resistivity, flexural strength, superplasticizer, acoustic emission, ultrasonic energy

## Abstract

The present article investigates the effect of superplasticizer and graphene nanoplatelet addition on the flexural and electrical behaviour of nanocomposites for applications related to the restoration/conservation of Cultural Heritage Monuments in laboratory scale. Graphene nanoplatelets’ addition is used to transform the matrix into a piezo-resistive self-sensor by efficiently dispersing electrically conductive graphene nanoplatelets (GnPs) in the material matrix to create electrically conductive paths. Nevertheless, the appropriate dispersion is difficult to be achieved as the GnPs tend to agglomerate due to Van der Waals forces. To this end, the effect of the addition of carboxyl-based superplasticizer (SP) is proposed in the present investigation to efficiently disperse the GnPs in the water mix of the binders. Five (5) different ratios of SP per GnPs addition were examined. The GnPs concentration was chosen to be within the range of 0.05 to 1.50 wt.% of the binder. The same ultrasonic energy was applied in all of the suspensions to further aid the dispersion process. The incorporation of graphene nanoplatelets at low concentrations (0.15 wt.%) significantly increases flexural strength when added in equal quantity to superplasticizer (SP1 series). The SP addition at higher concentrations does not enhance the mechanical properties through effective dispersion of the GnPs. Additionally, a correlation was established between the electrical resistivity (ρ) values of the produced nanocomposites and the modulus of elasticity as a function of the GnPs concentration. The functional correlation between these parameters was also confirmed by linear regression analysis, resulting from the experimental data fitting. Finally, the acoustic emission (AE) can effectively capture damage evolution in such lime-based composites, while the emitted cumulative energy rises as the GnPs concentration is increased.

## 1. Introduction

Structural health monitoring (SHM) is crucial for evaluating the performance of buildings and infrastructure of cultural heritage. It is important that both monitoring equipment and intervention materials are non-noticeable and non-intrusive, ensuring mechanical compatibility and preserving aesthetics. Thus, designing smart sensors in conservation engineering, consisting of compatible restoration materials, offers a promising integrated solution for monitoring and improving the safety of historic structures [1]. Typically, smart sensors undergo reversible changes in their electrical resistivity that occur in response to variations in stress and strain, along with irreversible changes in electrical resistivity caused by crack propagation.

The selection of the building materials of this article considered the environmental impact of the CO_2_ emission by the cement industry [2] and the compatibility potential between them and the original building [3]. The addition of metakaolin (MK) results in reduced consistency and increased cohesiveness in concrete, facilitating easier pumping and placement without bleeding [4]. The addition of metakaolin is oriented towards the enhancement of flexural strength, as has been proved in historic buildings [5]. The mineral composition of lime–MK mortars led to the quick formation of hydraulic products within the first few days of curing, demonstrating rapid hardening and high initial mechanical strength [6]. As the lime comes in contact with water, it starts dissolving, producing OH- and Ca^2+^ ions [7]. Metakaolinite-rich materials are currently among the most effective industrially produced pozzolans. These materials are created by calcining kaolinitic clays or claystones and then milling the resulting calcinates. In the presence of water, metakaolinite can react with calcium hydroxide (a pozzolanic reaction) to produce calcium silicate hydrate gel (CSH), stratlingite (C_2_ASH_8_), tetracalcium aluminate hydrate (C_4_AH_13_), and portlandite (CH) [8].

Cement and pozzolans are non-conductive building materials i.e., they do not possess electrical properties (self-sensing ability), and therefore, a reinforcement at the nanoscale must be incorporated to provide them with reduced electrical resistance. Carbon-based nanomaterials (CBNs), particularly carbon nanotubes (CNTs) and graphene nanoplatelets (GnPs), have been thoroughly studied for application in construction materials due to their extremely small geometrical dimensions, i.e., 1 to 20 nm [9]. The nano-reinforcement leads to controlled dissemination of cracks at the nano scale that are analysed through the Linear Elastic Fracture Mechanics (L.E.F.M.) theory [10]. The mechanical performance of cement pastes with graphene nanoplatelets has been extensively studied mainly assessing their performance under compressive mechanical loads. Focus of this article will be given in bending tests aiming to clarify the brittle failure of the investigated pastes.

Superplasticizers improve the workability by significantly increasing the fluidity of the cement mix without additional water, making the mixture easier to work with and place, especially in complex forms or structures with dense reinforcement [11]. In some cases, the mechanical strength is increased due to the denser packing of particles and reduced porosity. Additionally, they can accelerate the very early hydration process [12], leading to quicker mechanical strength development, less shrinkage and fewer cracks as concrete cures. In the last years, SP incorporation has been examined in grouts in masonry and historical buildings, e.g., [13,14]. In a recent study, with the exploitation of a superplasticizer and 60 min application of ultrasonic energy, the dispersibility of graphene nanoribbons in water increased by 25.7% in alkaline solutions [15]. Despite the interest in the use of SP and carbon-based nanomaterials in ternary matrices, there is still minimal research, and there are no relative research papers on the combined use of GnPs and SP in restoration lime-based pastes.

When a material undergoes mechanical stress, it can lead to the release of energy in the form of elastic acoustic waves [16]. These waves travel through the material and can be detected by specialised sensors or transducers to extract valuable information about the material or structure. Among the researchers that have investigated the mortars applied in Cultural Heritage Monuments by means of acoustic activity are Livitsanos et al. [17], who investigated the impact of wave propagation in masonry components to establish a strong correlation between propagation characteristics and material properties. Grazzini et al. [18] concluded that the Acoustic Emission (AE) technique permits the estimate of the amount of energy released in the de-bonding surface between repair mortar and stone during the damage process. Ospitis et al. [19] indicated that AE behaviour serves as an indicator of mechanical performance long before damage. Kalteremidou et al. [20] investigated the capability of AE to identify the dominant stress/strain component rather than solely detecting the occurrence of damage modes, particularly during the initial stages of loading. Therefore, there is a need for AE finding’ extension in restoration materials, applied not only in masonry but also in the Monuments of the 20th Century.

This article focuses on the development of lime-based pastes with different concentrations of GnPs and superplasticizers to promote the self-sensing ability of the paste. Uniformly dispersed GnPs were studied with respect to the electrical and mechanical properties (behaviour under flexure). The method of acoustic emission was employed to study the early damage of the nanocomposites, investigating if it could be useful for the non-destructive evaluation of lime-based composites. Comparative analyses of flexural performance and electrical resistance measurements were performed, and equations were proposed for specific GnP concentrations.

## 2. Experimental Procedures

**Figure 1:**
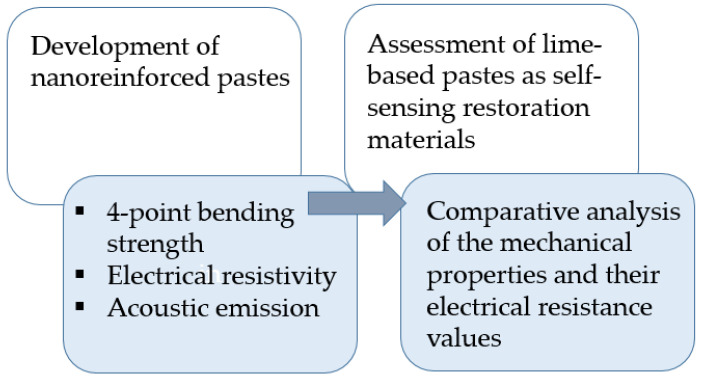
Schematic experimental process of the present study.

This section outlines the experimental methodology adopted in this study, which can be summarised as follows: (i) lime–metakaolin composite pastes were prepared, and (ii) mechanical and electrical resistance tests were conducted on the composite pastes. Additionally, acoustic emission (AE) sensors were attached to the specimens during the mechanical tests of the SP1 series pastes to trace the fracture intensity as this specific seriesexhibits the best mechanical performance compared to the other series without and also with higher amount of SP (as shown in Section 3.2). The experimental methodology is graphically illustrated in Figure 1.

### 2.1. Materials—Preparation of Lime-Based Nanocomposites

The examined binder at concentrations of 35 wt.% hydrated lime (L) with brand name (CaO Hellas), made in Greece, 35 wt.% metakaolin (MK) (ArgicalTM-M1000), made in France and 30 wt.% cement (CEM) (Aalborg White^®^ CEM I 52.5 N), made in Denmark will be referred to hereafter as ternary paste. The aqueous solutions of nanomaterials contain bottled water (Vikos S.A.), superplasticizer (SP) (ViscoCrete^®^ 5600 HS), made in Greece and graphene nanoplatelets (GnPs) type N008-100-P40 (Angstron Materials Inc.^®^, Dayton, OH, USA) and were dispersed at the optimum ultrasonic energy of 65 kJ energy following [21]. The GnPs had, according to the manufacturers’ data sheet, an average thickness of approximately 50 to 100 nm, a typical surface area of 15 m^2^/g and an average particle diameter less than 44 µm. Polycarboxylate superplasticizers typically have a backbone with hydrophilic carboxylate groups and hydrophobic or aromatic side chains. The hydrophobic side chains can interact with the sp^2^ regions of graphene via π-π stacking or van der Waals forces [9].

The GnPs were added at 0.05 wt.%, 0.15 wt.%, 0.20 wt.%, 0.25 wt.%, 0.50 wt.%, 1.00 wt.%, 1.50 wt.%, by weight of the solids (L+MK+CEM). Five (5) different ratios of superplasticizer to nanomaterial concentration were examined (namely 0, 1, 2, 4 and 8) that hereafter will be named as SP0 series, SP1 series, SP2 series, SP4 series and SP8 series, respectively. Each nanocomposite will be referred to as a compound word, in which the first part will denote the SP concentration, and the second one will symbolise the GnPs concentration expressed as a weight percent of solids (e.g., 0.30SP_0.15GnPs means that the paste consists of 0.30 wt.% SP and 0.15 wt.% GnPs). In Table 1, the nanocomposites on which both the mechanical and electrical properties were studied (in Section 3.3) are presented. Following other researchers who studied the effect of SP in cement mortars [22,23] in natural hydraulic lime-based pastes [24] and in aerial-lime pastes [25,26], the above ratios were determined. The reference paste (Table 1) contained neither SP nor GnPs. In addition, a complete experimental batch was used as SP reference (0.15SP_0.00GnPs, 0.30SP_0.00GnPs, 0.60SP_0.00GnPs, 1.20SP_0.00GnPs) in which the pastes did not contain any nanomaterials.

According to the literature, the high surface area of nanomaterials requires additional water to wet their surfaces, which in turn reduces the amount of free water available for moistening the ternary paste at a given water-to-cement ratio (w/c) [27]. To determine the appropriate water-to-binder (w/b) ratio, the ASTM C1437 standard; Test Method for the Flow of Hydraulic Cement Mortar, West Conshohocken, PA, USA, 14 October 2020 [28] was followed. The table had a diameter of 300 mm. The material was placed in a truncated cone mould having a height of 60 mm and a top diameter of 70 mm. The mould was lifted, and the mix was expanded, dropping the table for 25 repetitions over approximately 15 s as proposed by [29]. Five different w/b ratios were tested, ranging from 0.50 to 0.80 with 0.05 increments. The acceptable limits for flow were between 100% and 115%, calculated as a percentage of the cone base (100 mm). In each paste, the flow was measured. According to the results, the optimum w/c ratio was determined to be 0.55. Del Moral et al. [30] suggested a ratio of 0.5, and Jaafri et al. [31], a ratio of 0.6. Nevertheless, pastes with high SP content (ratio SP/GnPs = 2, 4 and 8) and GnPs concentration equal to 0.50 wt.%, 0.25 wt.%, 0.15 wt.% and 0.25 wt.%, respectively, namely (1.00SP_0.50GnPs, 1.00SP_0.25GnPs, 1.20SP_0.15GnPs and 2.00SP_0.25GnPs) were ultra-high flow pastes and the flow table test could not be conducted. Therefore, the ratio w/b was kept constant, thus eliminating the effects of inconsistency between the pastes.

**Figure 2:**
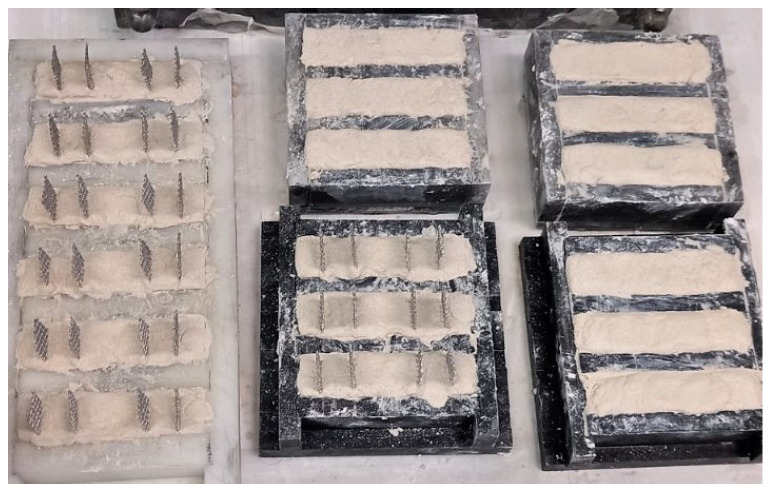
Image of the casted specimens in moulds, prismatic for flexural tests (right) and prismatic with embedded grids for resistivity tests (left).

After mixing, the pastes were cast, moulded and stored for 48 h at room temperature, as seen in Figure 2. After 48 h, all specimens were demoulded and placed in water tanks, fully immersed in saturated calcium hydroxide (0.82 g/L Ca (OH)_2_ solution) [32]. Generally, the specimens are cast into moulds for about 24 h regarding both the cement [33,34] and lime pastes [29,35]. In this research, in order to prevent microcrackings during demoulding, especially for the ternary pastes with high SP content, the samples were demoulded at 48 h, following other researchers [36]. Twelve (12) prismatic specimens with dimensions 80 mm in length, 20 mm in width, and 20 mm in height were prepared for each paste. Six (6) specimens of them were produced with embedded mesh stainless steel electrodes, as shown in Figure 2, to facilitate the electrical resistance measurements. During the casting process, four steel electrodes were inserted into the specimens, extending across the entire cross-section. The spacing between the outer and inner electrodes was 15 mm, while the gap between the inner electrodes measured 30 mm [37]. All the specimens were cured for 28 days.

### 2.2. Electrical Resistance Measurements

The widely used method to evaluate the electrical properties of materials is the four-probe technique. Four-probe measurement works by contacting four equally spaced probes to a conductive surface. Between probes 1 and 4, a current is applied, and the voltage drop between probes 2 and 3 is measured. Following Ohm’s Law, the electrical resistance and resistivity, which considers the geometrical features of the specimens, are calculated [38].

**Figure 3:**
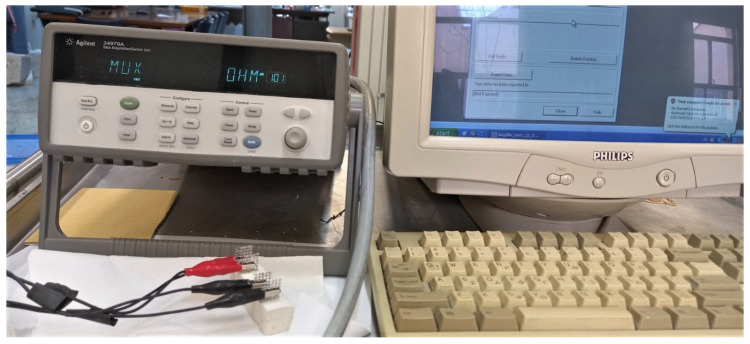
Image showing the four-point electrical measurement of a prismatic specimen, the electrical measurement device and the data acquisition system.

The electrical resistance of the pastes under Direct Current (DC) current was measured using prismatic specimens with the configuration shown in Figure 3. Ambient laboratory conditions typically falling within the following ranges: temperature: 20–25 °C (68–77 °F) and relative humidity (RH): 40–60%, following other researchers [39], were met. These conditions are generally maintained to ensure stability in experiments, minimise moisture-related effects, and prevent unwanted reactions in materials. Resistivity measurements were carried out on six (6) specimens, and the average values for each batch of GnPs-paste composites were calculated. To measure the electrical resistivity of the samples, metallic grids with openings (1.74 mm × 1.74 mm) were utilised as electrodes and embedded in the specimens right after casting, as previous researchers indicated [40]. All the specimens were covered with a membrane and left undisturbed until demoulding after 48 h. Once demoulded, the samples were cured in lime-saturated water for 28 days, followed by drying in an oven at 80 °C for three days to evaporate the water trapped in the pores, following the methodology [41]. This drying process was intended to minimise the impact of polarisation and was also selected over vacuum drying.

Vacuum drying can significantly impact the structure of pastes. Deep dehydration at elevated temperatures (above 100 °C) can lead to the breakdown of the hydration phases (e.g., calcium silicate hydrates, calcium hydroxide and ettringite) in lime–cement pastes, causing shrinkage and cracking. Additionally, the removal of water from gel pores and capillary pores can also cause microcracking and increased porosity, negatively affecting the mechanical integrity of the composite [42]. The four-probe method was applied, with a Data Logger Keysight Technologies^®^ 34970A, Santa Rosa, CA, USA was used for the measurements as shown in Figure 3, also reported by Dimou et al. [43].

### 2.3. Mechanical Testing

Prismatic specimens were subjected to four-point bending (flexural) tests conducted using a 10 kN VTS testing machine, following ASTM C78 standards, West Conshohocken, PA, USA, 1 March 2022 [44]. The experiment was conducted at a constant crosshead displacement rate of 0.02 mm/min to achieve quasi-static conditions. The loading span was kept at half the length of the support span.

Following the Euler–Bernoulli Beam Theory, the Modulus of Elasticity (MoE) was calculated. This foundational theory for bending analysis in beams assumes that beam sections remain plane and perpendicular to the neutral axis after deformation (i.e., no shear deformation, only bending). The term “nominal bending stress” refers to the bending stress calculated using the Bernoulli–Euler beam theory, which assumes a linear relationship between bending moment, material deformation, and stress distribution across the beam’s cross-section. It is computed using the “active” cross-sectional area, meaning the portion of the cross-section that directly resists the applied loads while ignoring any stress concentration effects, such as those introduced by notches, holes, or geometric irregularities [45]. The formula used in the 4-point bending test is derived from the relationship between the applied force and the resulting deflection in a beam subjected to bending.

(1) $$E=F\cdot a\cdot \left(3\cdot L^{2}-4\cdot \alpha^{2}\right)/48\cdot I\cdot \delta ,$$

where *E* = modulus of elasticity (MPa); *F* = applied force (N); *a* = distance from supports to load points (mm); *L* = span length (distance between supports) (mm); *I* = second moment of area (moment of inertia) (mm^4^), which depends on the cross-sectional shape. For a rectangular cross-section: *I* = *b*·*h*^3^/12, where *b* = width and *h* = height of the specimen and *δ* = measured deflection at the midpoint (mm).

Eventually, if the material behaves linearly and exhibits small deflections in the elastic range (i.e., there is no significant plastic deformation or nonlinear behaviour), it may be acceptable to use the deflection recorded by the testing machine. In this case, the deflection is small enough that the machine deflection and the actual deflection of the specimen are approximately the same. When dealing with small deflections, linear elastic materials, and well-calibrated test equipment, using the testing machine deflection can give a reasonably good estimate of the modulus of elasticity, Equation (1). On the contrary, when the deflections are large, high-precision applications, or setups where the machine’s own deflection could distort results, using an extensometer is necessary for accuracy.

### 2.4. Acoustic Emission Mearurements

**Figure 4:**
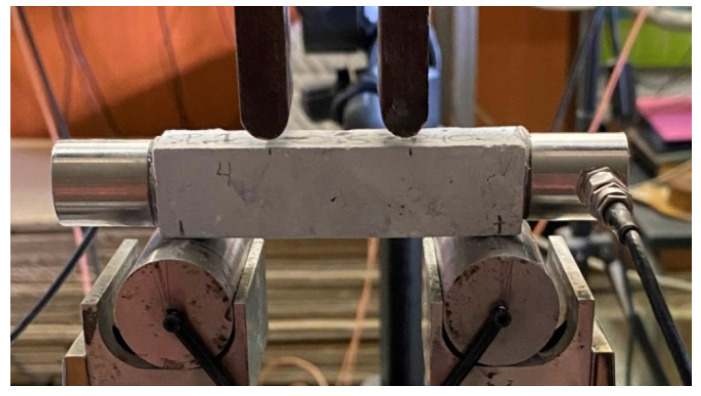
Image of the experimental flexural test (four-point bending) with two cylindrical R15a acoustic sensors attached at the lateral surfaces of the prismatic specimen.

One of the studied groups, more specifically the SP1 series, was selected to be investigated in-depth by exploiting the Acoustic Emission (AE) sensors, since they gave the most promising results in terms of mechanical performance (Section 3.2). The specimens were subjected to four-point bending (as it was described in the previous sub-section) and their acoustic activity was recorded by means of two R15α acoustic sensors (Mistras Group, Inc., Princeton Junction, NJ, USA) attached with the aid of silicone at the two lateral surfaces of each specimen, as seen in Figure 4, to facilitate effective acoustic wave transmission between the sensor and the surface being monitored. AEwin64™ software analyses AE hit data to mathematically calculate the location of all AE events. The R15α sensors typically operate at a resonant frequency of 150 kHz, making them ideal for detecting acoustic signals in this range. The tests were carried out quasi-statically, and the displacement-control mode was at a constant rate equal to 0.02 mm/min.

## 3. Results and Discussion

### 3.1. Electrical Characterisation

**Figure 5:**
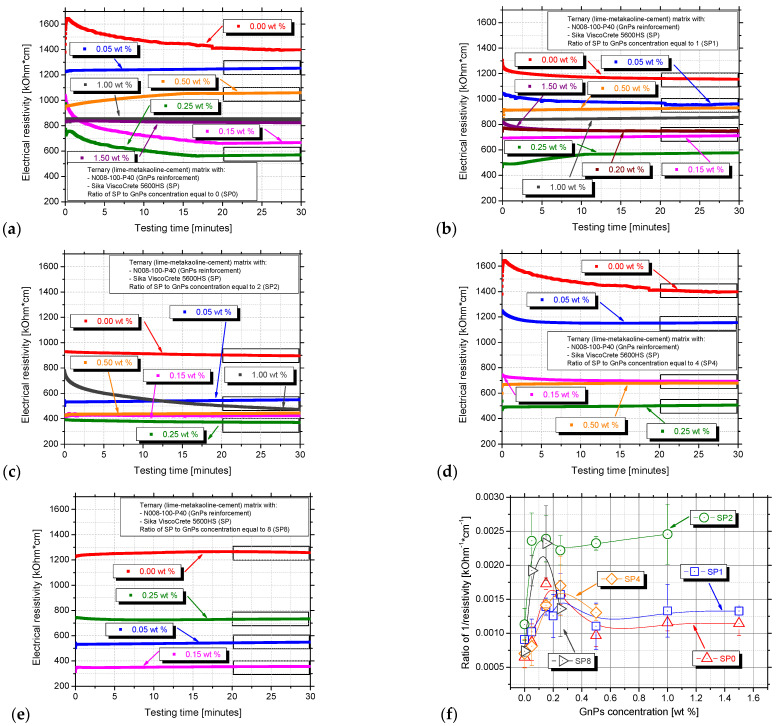
Electrical resistivity measurements of nanocomposites with different SP/nanomaterial ratios: (**a**) SP0, (**b**) SP1, (**c**) SP2, (**d**) SP4 and (**e**) SP8. Diagram (**f**) shows the reciprocal of average calculated resistivity values (1/ρ) determined with varying GnPs and SP concentration.

Figure 5 displays the typical electrical resistivity curves over time for plain cement paste and nanocomposites dispersed using ultrasonic energy with and without superplasticizer. For the reference paste of each series, high electrical resistivity values, around 1.4 MOhm·cm, were observed in the SP0 and SP4 series and approximately 1.2 MOhm·cm in the SP1 and SP8 series. The resistivity of the reference paste is similar to [46], who studied lime–cement mortars regardless of their lime content. The 0.30SP_0.00GnPs (SP2 series) paste exhibited a lower resistivity of 900 MOhm·cm. At the beginning of the test, resistivity values were higher, even 1.65 MΩhm·cm, likely due to the electrical polarisation effect. Electric polarisation occurs when the centres of positive and negative charges do not align, typically under the influence of a DC electric field, causing the material’s resistance to fluctuate, especially at the beginning of the measurements. This polarisation effect was also prominent in the samples 0.00SP_0.15GnPs and 0.00SP_0.25GnPs (shown by the pink and green curve), as well as in the 2.00SP_1.00GnPs (represented by the grey curves). After a few minutes of testing, the resistivity stabilised, and the electrical resistivity of the samples was determined as the mean value of the electrical resistivity values during the last five minutes (25 to 30 min) of testing.

All the nano-reinforced specimens exhibited lower electrical resistivity compared to the reference paste of each series. As the GnPs concentration is increased, the resistivity is expected to decrease, as previously investigated [47]. As more GnPs are incorporated into the matrix, the distance between neighbouring particles decreases, facilitating the formation of conductive pathways and leading to a significant reduction in the composite’s resistivity. The introduction of GnPs up to 0.05 wt.% was confirmed to reduce the resistivity of cement from 18.85 kΩ·m to 6.26 kΩ·m in the research of Guo et al. [48]. A similar trend is observed in the SP2 and SP8 series in the same GnPs content, leading to a −52.02% and 61.64% reduction, accordingly. Generally, at low GnP concentrations, the distance between individual GnPs is relatively large, resulting in the formation of a limited conductive network [38]. As a result, the resistivity of the matrix primarily determines the overall conductivity of the composite material in the 0.05 wt.% pastes of SP0, SP1, SP2, and SP4 series, Figure 5a–d. The 1.20SP_0.15GnPs paste exhibited 350 MOhm·cm, which is the lowest value of this concentration in all series, Figure 5e. That happens as extra superplasticizer can improve the plasticity of the matrix and decrease its friction with the GnPs [38]. Lower friction means better GnPs mobility within the matrix, allowing them to rearrange more efficiently and form conductive pathways. The paste with 1.20 wt.% SP (SP8 series) in Figure 5f exhibits the lowest value of resistivity that also comes in agreement with other researchers [49]. Also in the same graph, it is apparent that as the graphene concentration is further increased beyond the percolation threshold, the resistivity is decreased, mentioned also in [50]. The determination of the percolation threshold at 0.15 wt.% will also arise from the flexural tests in the next section.

### 3.2. Mechanical Properties

**Figure 6:**
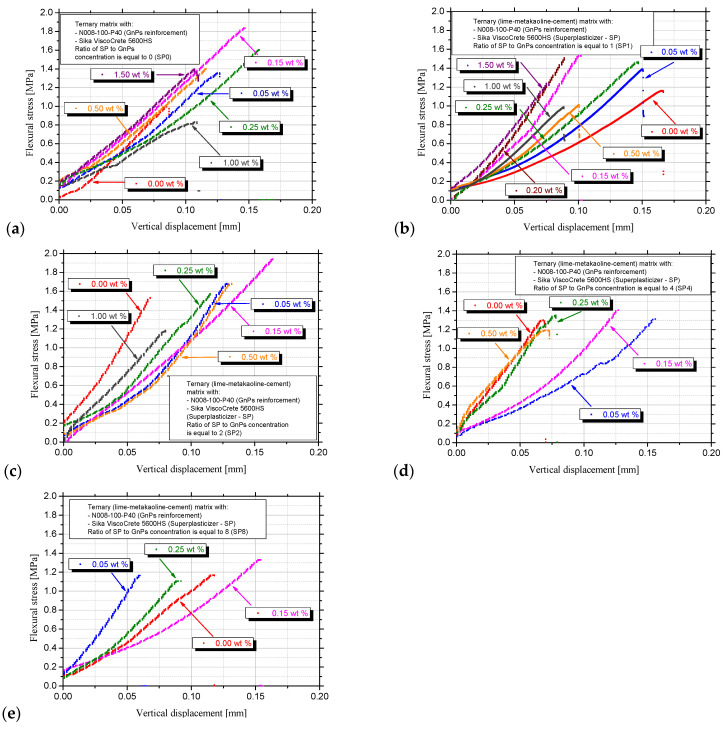
Characteristic curves of the flexural stress–vertical displacement from (**a**) SP0, (**b**) SP1, (**c**) SP2, (**d**) SP4 and (**e**) SP8 series.

All nanocomposite specimens under flexural loading exhibited a monolithic failure at the centre, indicating a compatible failure mode as confirmed by [51] in cement with SP. As the displacement at fracture is a reliable indicator of the material’s deformability, the four-point bending stress is plotted against vertical displacement. In Figure 6a,c,d, it is apparent that the displacement in which the fracture occurs in the pastes with 0.15 wt.% in the SP0, SP2, SP8 series, namely 0.00SP_0.15GnPs, 0.30SP_0.15GnPs and 1.20SP_0.15GnPs, is equal or greater than 0.15 mm. In Figure 6a–c, it is apparent that the displacement in which the fracture occurs in the pastes with 1.00 wt.% in the SP0, SP1, SP2 series namely 0.00SP_1.00GnPs, 1.00SP_1.00GnPs and 2.00SP_1.00GnPs, is lower than 0.10 mm, thus indicating the brittle nature of the high GnPs concentration specimen’s fracture as the material undergoes very little deformation before it breaks. In all flexural stress diagrams apart from Figure 6e, the maximum strength in the 0.05 wt.% paste is localised in displacement 0.12 to 0.16 mm, providing high energy absorption.

**Figure 7:**
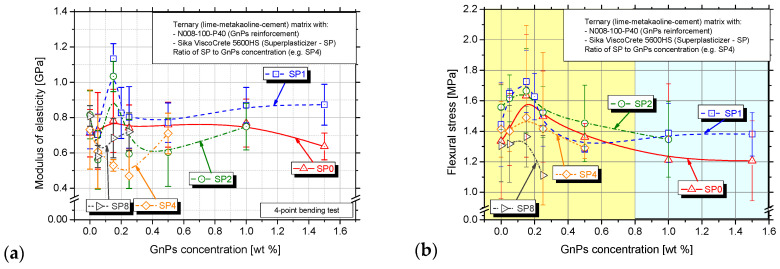
(**a**) Average values and standard deviation of modulus of elasticity and (**b**) flexural strength for the different investigated GnPs and SP concentrations.

In Figure 7a, the average values and standard deviation of the modulus of elasticity are presented. It is observed that MoE follows the same trend with the flexural strength (Figure 7b) except from the SP4 and SP8 series, where the MoE is decreased as the GnPs increase even when reaching the percolation threshold. Higher MoE means more resistance to bending stress, and that is observed in the percolation threshold (0.15 wt.%) in the SP0, SP1 and SP2 series. The trend observed in the SP1 series’ MoE is similar to that of Danoglidis et al. [33], where the nanocomposites with 0.10 wt.% GnPs presented the highest MoE. Additionally, a lower MoE denotes more deformation under the same stress.

In all the investigated series, almost the same pattern was observed regarding the variance of flexural strength, showing a peak value at 0.15 wt.% GnPs regardless of the SP concentration, Figure 7b. First, the initial values in the pastes without GnPs were similar between the SP0 and SP8 series, approximately equal to 1.3 MPa and between the SP1 and SP4 series, equal to 1.4 MPa. The 0.30SP_0.00GnPs (SP2) presented the highest flexural strength (1.56 MPa) in the series without nanomaterial. As the GnPs concentration is increased, the flexural strength is increased as well, reaching its maximum value at a GnPs concentration of 0.15 wt.% in all series, and then the flexural strength is reduced in series SP2, SP4 and SP8 and holds a stable value in the SP0 series. This means that the further addition of GnPs is no longer beneficial for the flexural strength. There is one GnPs concentration in all series above, in which the addition of GnPs does not enhance the flexural strength. Specifically, this is the 1.00 wt.% in SP0, 0.50 wt.% in the SP1 and SP2 series, 0.25 wt.% in the SP4 and 0.15 wt.% in the SP8 series. In the SP1 series, there is a slight increase after the 0.50 wt.% concentration.

For the explanation of the performance of the pastes, the main reactions of the binding material are presented below with the starting point of the cement hydration. The main hydration products of cement are ettringite (AFt), monohydrate aluminum sulfate (AFm), calcium hydroxide (CH) and hydrated calcium silicate (C-S-H) gel [9]. The portlandite (Ca(OH)_2_) dissolves in water, releasing Ca^2+^ and OH^−^ ions into the system. This increases the pH value of the solution (~12 to 13), providing an alkaline environment necessary for C-S-H formation. When lime (CaO) is added, it reacts with water to form more Ca(OH)_2_, which further dissolves to release Ca^2+^. This reaction ensures an abundant supply of Ca^2+^, but excess Ca^2+^ can cause flocculation of graphene nanoplatelets (GnPs) and cement particles. Metakaolin (MK) does not directly release Ca^2+^, but it reacts with Ca(OH)_2_ in a pozzolanic reaction, reducing the free Ca^2+^ in the system and producing calcium alumino-silicate hydrates [52], as:

2C_3_S + 6H_2_O→3C-S-H + 3Ca(OH)_2_

2C_2_S + 4H_2_O→C-S-H + Ca(OH)_2_

CaO + H_2_O→Ca(OH)_2_ and Ca(OH)_2_→Ca^+2^ + 2OH^−^

Al_2_O_3_⋅2SiO_2_ + Ca(OH)_2_ + H_2_O→C-A-S H.

The SP addition significantly delays cement hydration and setting time by adsorbing onto cement particles, which restricts the movement of ions and water [53]. As hydration advances, the SP presence raises the hydroxide concentration in the cement pore solution [15]. Excessive SP quantity can cause possible separation of the cement particles, leading to segregation. Some SP particles create a thin water layer around these particles, but in excess, it leads to excessive bleeding (water rising to the surface), making the paste sticky and reducing its ease of placement [54]. Properly dispersed GnPs can possibly nucleate additional C-S-H (calcium silicate hydrate) formation, strengthening the cement structure, improving mechanical strength, reducing porosity, and enhancing durability [55].

**Figure 8:**
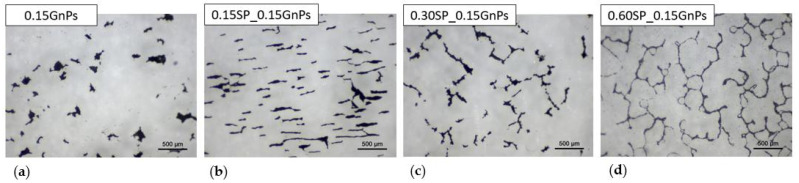
Optical microscopy images for the 0.15 wt% GnPs aqueous dispersion in (**a**) SP0, (**b**) SP1, (**c**) SP2, and (**d**) SP4 series dispersed applying a 65 kJ ultrasonic energy.

The effect of the SP addition on the dispersion of 0.15 wt.% GnPs in aqueous solution was observed by means of light optical microscopy (Figure 8). All the samples were ultrasonicated at 65 kJ ultrasonic energy to dissolve the GnPs agglomerates. Typically, the SP long polymer chains extend into the solution, which prevents GnPs from restacking following the steric hindrance mechanism. Polycarboxylate SP contains negatively charged carboxyl (-COO−) groups, which can create an electrostatic repulsion barrier between the GnP particles [56]. These -COO− groups dissociate in water, making the SP molecules negatively charged. GnPs have strong van der Waals forces, causing them to agglomerate. Unlike graphene oxide (GO), pure GnPs have fewer oxygen functional groups (-OH, -COOH, etc.) on their surface, making them less dispersible in water-based systems. In alkaline cement environments, the -COOH groups deprotonate to form -COO−, making GnPs negatively charged. Since both, SP and GnPs are negatively charged (-COO− groups), they repel each other due to Coulomb’s law. The carboxyl and hydroxyl functional groups are thought to chemically bond with the two primary cement hydration phases—hydrated calcium silicate (C-S-H) and calcium hydroxide Ca(OH)_2_ [23]. If the bonding alters the pore structure (e.g., refining or reducing connectivity of pores), it might increase resistivity by limiting the movement of free ions in the cement paste. Without repulsion, GnPs would cluster together, reducing their reinforcing effect in cement, as observed in Figure 8a in the 0.15GnPs solution with the absence of SP. When an equal quantity of SP is added (SP1), well-aligned arrays of elongated GnP particles are developed, which can be seen in Figure 8b. In 0.30SP_0.15GnPs solution (referred to as SP2 in Figure 8c), the conductive paths provoke denser microstructure and lower porosity, which endows the sample with lower electrical resistance or higher ratio 1/ρ as it is in agreement with electrical measurements in the SP2 series in Figure 5f. Finally, in the 0.60SP_0.15GnPs dispersion (SP4 series in Figure 8d), a continuous network of conductive paths is formulated. When a continuous network of conductive paths is formed, the enhanced ion mobility—due to the addition of SP—improves the ionic conductivity of the system. This occurs because SP disperses cement grains, releasing trapped water and increasing the availability of free ions, which facilitates better ionic movement. This, in turn, enhances the interaction between GnPs and the pore solution, complementing the electronic conductivity of GnPs, as also indicated in [49].

While moderate SP dosage helps water reach cement particles by dispersing them, an excessive amount of SP can block water access (over-adsorption effect), delaying hydration. Therefore, in cement systems, Ca^2+^ can cause flocculation first, but if uncontrolled, it leads to aggregation that is stronger and irreversible in contrast to flocculation. Superplasticizers help prevent flocculation and stop it from turning into aggregation [57]. Electrostatic repulsion from SP ensures that both cement particles and GnPs remain well separated, leading to better workability. Calcium ions (Ca^2+^) from cement can neutralise the negative charges on both SP and GnPs, reducing repulsion. At high concentrations, superplasticizer molecules form multiple layers on the nanoparticle surface. Consequently, some surfactant molecules extend into the liquid phase and begin to interact with each other. This interaction leads to the flocculation of nanoparticles, negatively affecting dispersion [58]. This is why the SP8 series is observed to reduce the mechanical properties creating flocs, decreasing the efficient dispersion of GnPs. Therefore, a balance of superplasticizer dosage is crucial to maintaining repulsion while controlling Ca^2+^ interactions.

As the GnPs concentration is increased, the flexural strength is increased as well, reaching its maximum value in the pastes with 0.15 wt.% GnPs in all series, and then the flexural strength is decreased and holds a stable value. That means that the further addition of GnPs is no longer beneficial for the flexural strength. That is obvious in the SP0 and SP1 series. In Figure 7b, it is obvious that the 0.15 wt.% concentration consists of the percolation threshold where an interconnected network of hydration products forms, providing structural integrity and significant development of mechanical strength. Similar results indicated the same concentration between 0.10 and 0.25 wt.% in cementitious nanocomposites with GnPs [59]. Pivák et al. [60] also proved that the excellent graphene properties were exploited even when the lowest dosage of a GnPs (0.10 wt.% to the binder) was applied in lime-pastes.

Regarding the SP0 series, the 0.00SP_0.15GnPs shows a +22.26% increase in flexural strength compared to the reference paste, and this enhancement agrees with Wei et al. [61], who proved that cement mortar enhanced with 0.10 wt.% graphene nanofluid additives exhibited improved flexural strength (+24.72%). By identifying and optimising the percolation threshold, engineers can tailor lime-based materials for advanced applications such as smart nanocomposites and enhanced durability. The exceptional properties of graphene can be utilised effectively even with the minimal addition of the nanomaterial (0.15 wt.% relative to the binder). The 0.00SP_0.05GnPs paste exhibits a +5.68% increase in flexural strength compared to the reference paste that complies with Khan et al. [62] that added 0.03 wt.% GnPs to a reference sample without graphene, and the sample’s strength increased by +5.04%.

In the 0.15 wt.% GnPs concentration by adding an equal quantity of GnPs and SP, the flexural strength was increased by +5.69% compared to the SP0 series. In the SP4 and SP8 series, the decline was −8.7% and −16.4%, accordingly. In the 1.00 wt.% GnPs concentration by adding an equal quantity of GnPs and SP, the flexural strength was increased at +14.6% compared to the SP0 series, and when the SP was double to the GnPs, the flexural strength increase was +11.1%. The slight increase of the flexural strength in the 1.00SP_1.00GnPs is attributed to the flocculation. The water released from paste flocculation compensated for the water absorbed by the GnPs, thereby mitigating the adverse impact of GnPs on fluidity [9].

### 3.3. Correlation of the Physical Properties

**Figure 9:**
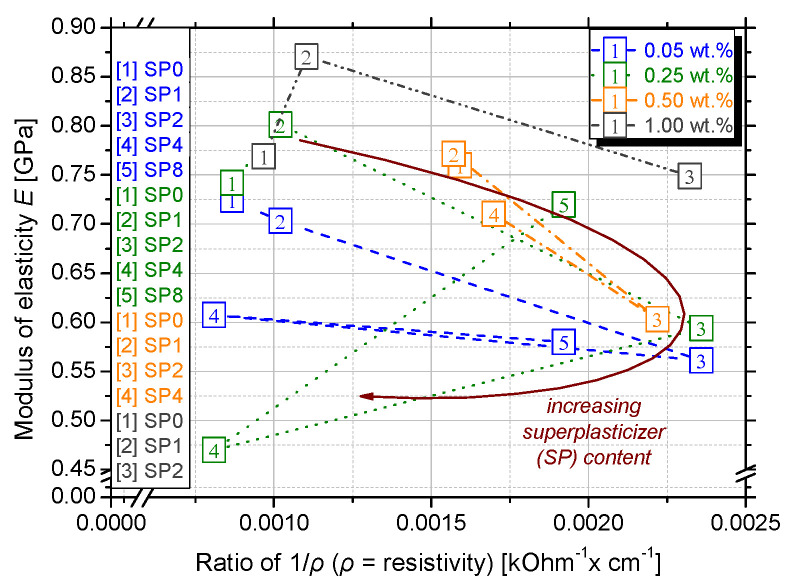
Average values of modulus of elasticity for the different investigated GnPs and SP concentrations.

Figure 9 shows the correlation between the average values of the modulus of elasticity for nanocomposites with 0.05 wt.%, 0.25 wt.%, 0.50 wt.% and 1.00 wt.% GnPs and conductivity (1/ρ). A remarkable pattern is observed regarding the above GnPs concentrations and their electrical performance as the SP content is increased. The maximum conductivity is noticed in the SP2 series, and then it is decreased again in the SP4 series and increased in the SP8, following a circular pattern. SP particles reduce the flocculation of cement particles by adsorbing onto their surface and providing electrostatic and steric repulsion [24]. This leads to a more homogeneous microstructure, reducing pore blockages and allowing for better connectivity of conductive paths. This explains the reduction of resistivity in the SP2 series. Nevertheless, excessive amounts may lead to excessive porosity or segregation, which could negatively impact mechanical properties, as it is clear in the SP4 series where the ρ is elevated again. The negative functional groups on superplasticizer molecules can increase ionic mobility by interacting with Ca^2+^, Na^+^, K^+^, and OH− ions in the pore water. Since resistivity is inversely proportional to ionic conductivity, higher ion concentration and mobility result in lower resistivity, as observed in the SP8 series [49]. On top of that, excess superplasticizer can delay C-S-H gel formation, keeping more free ions in solution and making the system more conductive [24].

The evolution of electrical resistivity is able to detect the development of microstructure [49] and, especially when the GnPs concentration is increased, the MoE is also enhanced. This could be because of the denser microstructure in higher GnPs dosages that leads to material resistance, therefore better deformation and increasing elastic modulus.

To correlate the damage extent with the measured electrical conductivity, linear regression analysis was applied in the experimental values of MoE and 1/ρ, which can be seen in Figure 9. That is identified with the 0.05 wt.%, 0.25 wt.%, 0.50 wt.%, and 1.00 wt.% GnPs concentrations while varying the SP content and the MoE in juxtaposition to resistivity was measured. The equation that associates the MoE and 1/ρ and describes their functional relation was proposed in all series—Equations (2) and (3). Investigation will be made to seek whether this correlation changes regarding the incremental SP concentration.

(2) $${\mathrm{MoE}}_{0.05\;\mathrm{wt}.\%}\;=-79.69922*\frac{1}{\rho }+0.74658,\;R^{2}=0.42518$$

(3) $${\mathrm{MoE}}_{0.50\;\mathrm{wt}.\%}\;=-121.99437*\frac{1}{\rho }+0.88502,\;R^{2}=0.93728$$

Dealing with multiple regression analyses, the coefficient of determination (R^2^) values are used to describe the relationship between stress and resistivity across different experimental sets with varied concentrations. In some cases, attempting to fit a linear model to data that have a non-linear relationship, a negative R^2^ arises. The linear model would not capture the true relationship well, leading to poor predictive performance. In the 0.25 wt.% and 1.00 wt.% experimental series, logarithmic or exponential fits may better describe the relationship between variables as the R^2^ was negative. In the 0.50 wt.% set, 93.7% of the variance is explained by the regression model. This suggests an excellent fit between MoE and resistivity for this particular experimental set as well as in the 0.50 wt.% series despite the moderate R^2^.

Yet, the above equation expresses certain material and experimental parameters, such as GnPs and superplasticizer type and concentration, lime, metakaolin and cement, w/c, dimensions of specimens, and specific experimental features. This means that the x-coefficient that is depicted in Figure 9, as well as the constant values of the fitting equations, are expected to change when different material properties are chosen. However, it is apparent that the resistivity and mechanical properties are straightforwardly linked and that this methodology can be easily implemented to evaluate the monitoring ability of lime-based nanocomposites.

### 3.4. Acoustic Emission

It is well known that during four-point bending tests, the failure/fracture of the specimen is expected to be located at the central part of the specimen, between the two loading points. Moreover, this type of testing is characterised by the formation of tensile cracks at the bottom surface of the specimen, which lead to the fracture of the specimen.

**Figure 10:**
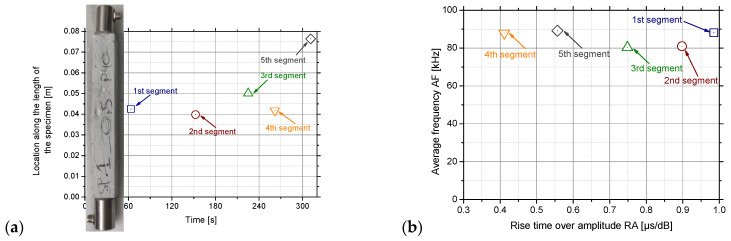
(**a**) The location of the acoustic events during the time of loading and (**b**) the AF versus the RA parameter for a typical specimen of the 0.50GnPs_0.50SP paste.

AE technique was employed in all specimens tested (the ones which were fractured close to the supports were excluded from the analysis). The exploitation of two acoustic sensors permitted the determination of the location of the damage (by means of the acoustic events produced) along the length of the specimen (i.e., linear location) during loading. The data of a typical specimen from the SP1 series, namely the 0.50GnPs_0.50SP paste, are used. The loading procedure up to the maximum load was divided into four segments (based on the instants where slope changes were observed at the load–time curve), and the fifth one refers to the decreased loading branch (after the maximum load). As a next step, the average location and the average time of loading were calculated, and they are presented in Figure 10a. Identifying the failure mode is crucial, as it offers valuable insights for optimising reinforcement design to effectively resist the specific stresses involved [63]. The localisation of damage at the central part of the specimen is obvious from the beginning of the experiment, and only after the failure of the specimen (creation of the main crack and decrease of load) are parasitic acoustic events produced close to one support. Afterwards, the average values of the parameters RA (i.e., Rise Time over Amplitude) and AF (i.e., Average Frequency) were calculated for the same five segments.

The AF vs. RA graph is a commonly used graph in order to qualitatively characterise the damage/cracks as tensile or shear/mixed ones [64]. As can be seen from Figure 10b, the average frequency of the AE remains equal to almost 85 kHz from the beginning of the experiment until the fracture of the specimen. This is in agreement with Livitsanos et al. [65], who proved the AF was equal to 73.31 in lime–cement mortars loading from 95 to 100%. This value is consistent with previous research where the incorporation of 0.40 wt.% GnPs and superplasticizer at a ratio of 1.5/1 in respect to graphene in cement mortars provoked a signal of AF around 72 kHz [66], thus allowing almost direct comparison with the SP1 series and mainly the 0.50GnPs_0.50SP paste. As the AF was consistently detected as almost stable in all investigated pastes at 80 kHz, it means that AE can effectively capture damage evolution in these materials. The method may be sensitive enough to track early damage, making it useful for the non-destructive evaluation of lime-based composites. On the other hand, the RA values become lower values as the experiment progresses and tensile cracks start to generate. This trend of AF vs. RA in a single specimen is also verified in concrete [67].

The abovementioned behaviour of the specimen was the typical one observed in all experiments. The concentration of the GnPs does not affect either the location of damage/failure or the tensile cracking mode, which are related to the macroscopic behaviour of the specimens. However, the GnPs concentration does affect some other acoustic characteristics. As it was probably expected, the higher the concentration of the GnPs, the higher the amplitude of the AE produced during loading [66]. More specifically, for the first three pastes (i.e., 0.05GnPs_0.05SP; 0.15GnPs_0.15SP; 0.25GnPs_0.25SP), the highest amplitude recorded was 70 to 80 dB, while for the 0.50GnPs_0.50SP and 1.00GnPs_1.00SP pastes, the maximum amplitudes detected ranged between 80 and 90 dB. Amplitudes higher than 90 dB were produced only for the 1.50GnPs_1.50SP paste. Similar AE amplitude values were recorded in cement composites reinforced with 0.04 wt.% GnPs, which touched a maximum limit of 70–80 dB during the peak stage [68].

**Figure 11:**
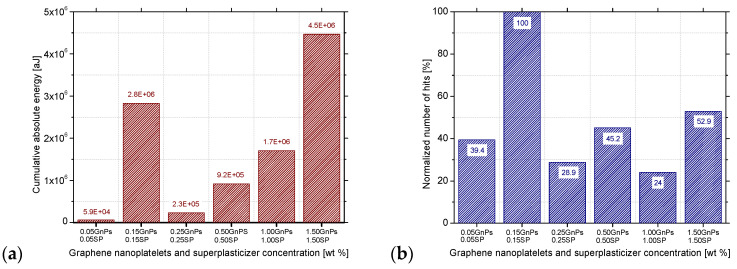
Acoustic emission results for the SP1 series and for various GnPs concentrations: (**a**) cumulative absolute energy hits and (**b**) normalised number of AE hits under flexural loading.

Nevertheless, this trend does not seem to be true when the absolute energy produced is studied. As it can be seen from Figure 11a, indeed, the minimum cumulative energy emitted is produced by the paste with the lowest GnPs concentration and the maximum cumulative energy emitted is produced by the 1.50GnPs_1.50SP paste, but the 0.15SP_0.15GnPs paste disturbs this increasing trend producing about half of the acoustic energy emitted by the 1.50SP_1.50GnPs paste. This trend is in line with previous researchers who also studied cement specimens reinforced with 0.1 wt.% up to 0.5 wt.% GnPs and substantiated a peak of cumulative energy in the 0.1 wt.% GnPs concentration 10 s before the failure [69]. This disturbance is related to the number of the acoustic hits produced during the loading of the specimens. More specifically, the number of hits produced by the 0.15SP_0.15GnPs paste is far larger than the number of hits emitted by the other groups, as shown in Figure 11b. From the same graph, the reduction in the number of hits between the 0.05SP_0.05GnPs and the 1.00SP_1.00GnPs is calculated at 15%, and there is a slight similarity with the hits reduction of the composites with 0.04 wt.% and 0.08 wt.% contents of GnPs over cement that was approximately 10% in the failure stage [68].

## 4. Conclusions

The present study investigates for the first time the impact of the SP in restoration pastes, relating the mechanical and electrical response. It is expected that the present study will build the foundation for the development of self-sensing restoration materials. The modification mechanisms are elucidated through a detailed analysis of flexural and electrical resistance measurements in the lime nanocomposites. The outcome can be summarised as follows:

- Considering its substantial effects on both electrical and mechanical properties, GnPs content at 0.15 wt.% seems to create the percolation threshold. When the paste is also mixed with SP, the best mechanical performance is attributed to an equal addition of SP. Excess use of SP is not beneficial.
- The electrical resistivity of the investigated pastes exhibited a notable configuration. Initially, as the content of GnPs increased (ranging from 0.05 wt.% to 0.15wt.% by binder), the resistivity decreased to 717.80 kOhms in the SP1 series. Nevertheless, for pastes incorporating up to 0.15 wt.% GnPs, a subsequent increase occurred till the 0.50 wt.% paste when an intermediate constant value was reached in the SP0, SP1 and SP2 series.
- A correlation between the modulus of elasticity and electrical resistivity was observed for the first time in lime-based pastes. The linear regression in 0.05 wt.% and 0.50 wt.% GnPs has comparable negative slope values in the above series of pastes with different amounts of SP. The SP content augmentation decreases the resistivity up to the SP2 series, and then the ρ is increased again in the SP4 series and decreased in the SP8, following a circular pattern.
- The AF equal to 80 kHz is consistently detected in the SP1 series, denoting that AE can effectively capture damage evolution in lime-based composites.
- The emitted cumulative energy rises as the GnPs concentration is also increased, except from the 0.15SP_0.15GnPs paste that produces about half of the acoustic energy emitted by the 1.50SP_1.50GnPs paste.

Consequently, this study analyses the combined integration of superplasticizer and GnPs in the flexural performance of lime-based nanocomposites and finally determines the actual relationship between the mechanical and electrical properties. Additional research is required in larger specimens to improve crack localisation by calibrating the AE travel speed for each GnPs concentration and employing multiple sensors to accurately determine crack positions within a three-dimensional space by considering longer propagation distances. As the correlation between MoE and electrical resistance is established in this article, the way is opened for measuring both the resistance and acoustic events at the same time. Following this methodology, the MoE will be measured by real-time digital image correlation that captures the full-field strain distribution and deformations on the specimen’s surface during the recording of acoustic events, which does not exist so far in the literature on lime pastes of specific dimensions. The long-term weather resistance of the piezoresistive sensors also needs to be considered for applications related to the restoration/conservation of Cultural Heritage Monuments in practical application.

## Figures and Tables

**Table 1:** Investigated nanocomposites of the present article.

Code Name of the Paste	SP Concentration (wt.%)	GnPs Concentration (wt.%)	Ratio SP/GnPs (-)
0.00SP_0.00GnPs (reference)	0.00	0.00	0
0.00SP_0.05GnPs	0.00	0.05	0
0.00SP_0.15GnPs	0.00	0.15	0
0.00SP_0.25GnPs	0.00	0.25	0
0.00SP_0.50GnPs	0.00	0.50	0
0.00SP_1.00GnPs	0.00	1.00	0
0.00SP_1.50GnPs	0.00	1.50	0
0.15SP_0.00GnPs	0.15	0.00	1
0.05SP_0.05GnPs	0.05	0.05	1
0.15SP_0.15GnPs	0.15	0.15	1
0.20SP_0.20GnPs	0.20	0.20	1
0.25SP_0.25GnPs	0.25	0.25	1
0.50SP_0.50GnPs	0.50	0.50	1
1.00SP_1.00GnPs	1.00	1.00	1
1.50SP_1.50GnPs	1.50	1.50	1
0.30SP_0.00GnPs	0.30	0.00	2
0.10SP_0.05GnPs	0.10	0.05	2
0.30SP_0.15GnPs	0.30	0.15	2
0.50SP_0.25GnPs	0.50	0.25	2
1.00SP_0.50GnPs	1.00	0.50	2
2.00SP_1.00GnPs	2.00	1.00	2
0.60SP_0.00GnPs	0.60	0.00	4
0.20SP_0.05GnPs	0.20	0.05	4
0.60SP_0.15GnPs	0.60	0.15	4
1.00SP_0.25GnPs	1.00	0.25	4
2.00SP_0.50GnPs	2.00	0.50	4
1.20SP_0.00GnPs	1.20	0.00	8
0.40SP_0.05GnPs	0.40	0.05	8
1.20SP_0.15GnPs	1.20	0.15	8
2.00SP_0.25GnPs	2.00	0.25	8

## Data Availability

Data are available upon request to the authors.
